# Spatial–temporal characterization of photoemission in a streak-mode dynamic transmission electron microscope

**DOI:** 10.1063/4.0000219

**Published:** 2024-02-23

**Authors:** Samik Roy Moulik, Yingming Lai, Aida Amini, Patrick Soucy, Kenneth R. Beyerlein, Jinyang Liang

**Affiliations:** Centre Énergie Matériaux Télécommunications, Institut National de la Recherche Scientifique, Université du Québec, 1650 Boulevard Lionel-Boulet, Varennes, Québec J3X1P7, Canada

## Abstract

A long-standing motivation driving high-speed electron microscopy development is to capture phase transformations and material dynamics in real time with high spatial and temporal resolution. Current dynamic transmission electron microscopes (DTEMs) are limited to nanosecond temporal resolution and the ability to capture only a few frames of a transient event. With the motivation to overcome these limitations, we present our progress in developing a streak-mode DTEM (SM-DTEM) and demonstrate the recovery of picosecond images with high frame sequence depth. We first demonstrate that a zero-dimensional (0D) SM-DTEM can provide temporal information on any local region of interest with a 0.37 *μ*m diameter, a 20-GHz sampling rate, and 1200 data points in the recorded trace. We use this method to characterize the temporal profile of the photoemitted electron pulse, finding that it deviates from the incident ultraviolet laser pulse and contains an unexpected peak near its onset. Then, we demonstrate a two-dimensional (2D) SM-DTEM, which uses compressed-sensing-based tomographic imaging to recover a full spatiotemporal photoemission profile over a 1.85-*μ*m-diameter field of view with nanoscale spatial resolution, 370-ps inter-frame interval, and 140-frame sequence depth in a 50-ns time window. Finally, a perspective is given on the instrumental modifications necessary to further develop this promising technique with the goal of decreasing the time to capture a 2D SM-DTEM dataset.

## I. INTRODUCTION

A suite of high-speed electron microscopy instruments and methods have been developed in the past few decades to provide unique views of the atomic processes involved in material excitations and dynamics.^1–3^ The most widespread representative of such instruments is the ultrafast transmission electron microscope (UTEM), which has been used to study metal-insulator phase transitions,^4^ expansion dynamics,^5^ anisotropic atomic structural dynamics of metal nanoparticles,^6^ magnetization dynamics,^7^ and biomechanical properties of DNA.^8^ This instrument uses ultraviolet (UV) light pulses from a high-repetition-rate ultrafast laser to generate low-charge electron pulses from a cathode material (< 10 electrons per pulse) via the photoelectric effect.^9^ As a single pulse does not contain sufficient electrons to form an image, a UTEM image is collected stroboscopically, and capturing one single frame can require an exposure time of 100 s.^1^ A time series of 20 frames can then take 30 min to collect, requiring stable microscope operation, no sample degradation, and consistency in its response on this time scale.

Alternatively, the dynamic transmission electron microscope (DTEM) has been developed for snapshot imaging and used to capture dynamics of many transient and irreversible processes, including the formation of thin films,^10,11^ intermetallic phase surface reactions,^12^ alloy melting,^13–16^ and rapid phase transformations.^17^ The DTEM relies on a high-intensity laser to generate high-charge electron pulses (∼10^7^–10^8^ electrons per pulse) that form an image in a single shot.^18,19^ At this limit, electron–electron interactions play a significant role in defining many properties of the electron pulses used in a DTEM. The maximum photoemitted current density is governed by the Child–Langmuir limit,^20,21^ the pulse energy distribution is broadened by the Boersch effect,^1^ and the pulse also suffers from longitudinal and transverse broadening at beam crossovers as it propagates in the column.^1^ Despite all these effects, no measurements of the temporal profile of high-charge electron pulses in a DTEM have been previously reported. Previous works have focused on characterizing low-charge photoemitted electron pulses used in UTEM instruments.^22–24^ In these studies, pulses containing up to 1000 electrons were found to have a pulse duration that was increased by 1 ps due to space–charge repulsion in the gun. The electron flux of these pulses (i.e., 500-electron/ps) is comparable with those generated in a DTEM, the difference being that the laser pulse used to generate an electron pulse in a DTEM is more than 20 000× longer, producing a proportionally larger total number of electrons in the pulse. Therefore, it is commonly assumed that the temporal broadening of a DTEM electron pulse is negligible compared to its pulse duration and that the temporal photoemission profile follows the UV pulse.

The movie-mode DTEM (MM-DTEM) has been developed to follow the dynamics of an individual transient event by capturing a sequence of images in a few microsecond time window.^25^ This operation requires the implementation of a nanosecond laser controlled by an arbitrary waveform generator to generate a customizable laser pulse train and a high-speed electrostatic deflector to deflect images formed by each pulse in the train to different regions of the TEM camera.^25,26^ However, the MM-DTEM's performance is limited to frame rates less than 100 MHz due to pulse durations longer than 10 ns and a delay between pulses to allow for stabilization of the plate voltage.^1^ Furthermore, as adjacent images recorded on the TEM camera should not overlap, a sequence of only 9–16 snapshots of an event can be captured with an MM-DTEM. Improving the DTEM frame rate and image sequence depth can allow new data acquisition strategies for imaging picosecond dynamics in a much shorter measurement time than possible with a UTEM. This improvement can alleviate the constraint that the sample and instrument must be stable for long acquisition times and allow for studies probing the ultrafast response of systems as they evolve on slower time scales. This ability could be useful to study materials that are especially prone to electron beam damage, such as mixed halide perovskites that are known to phase segregate and degrade when exposed to a moderate dose of a high-energy electron beam.^27–29^

Streak imaging is a popular technology for boosting temporal resolution and frame rate, which has been prevalent in optical imaging systems for the last few decades.^30–33^ By using an ultrafast temporal streaking unit that deflects an electron pulse to different spatial positions according to their arrival time, commercially available streak cameras have enabled direct measurement of transient signals with a temporal resolution as high as hundreds of femtoseconds.^34,35^ Recent years have witnessed substantial enhancements in the technical specifications of streak imaging systems, featuring broad sweep ranges spanning from picoseconds to milliseconds^36–38^ and high-dimensional data acquisition.^39,40^ Moreover, CCD/CMOS cameras with several million pixels have been incorporated as standard configurations to record streak images.^41^ These collective advancements empower streak imaging with the ability to operate across multiple imaging speeds, spanning from thousands to trillions of frames per second (fps).^42^

A few examples exist of how streak imaging has been adopted to enhance the temporal resolution of time-resolved TEM measurements. Streak imaging with a DTEM was first demonstrated by the Bostanjoglo group using nanosecond laser pulses with a sweep time of >20 ns.^43–46^ In this work, the approximate time scale of metal film melting was extracted based on the average contrast change in the streak images. This technique was applied to the study of hydrodynamic instability during melting with a micrometer-level spatial resolution and a tenth-of-microsecond-level temporal resolution.^43^ More recently, streak-mode (SM) imaging based on compressed sensing has been proposed to obtain picosecond temporal resolution and a nanometer spatial resolution in an MM-DTEM.^47^ This study showed the possibility of synergizing coded-aperture imaging and compressed-sensing-based image reconstruction via analytical modeling and numerical simulation to observe irreversible nanoscale dynamic events in real time at picosecond temporal resolutions.

This article presents our recent progress in implementing different modes of streak imaging functionality in an MM-DTEM. This involved changing the voltage waveform applied to the existing deflector plates, while keeping the electron optics and laser hardware of the instrument intact and unaltered. After detailing the instrumental setup, we demonstrate zero-dimensional (0D) SM-DTEM, which provides a direct measurement of image contrast changes in a specific nanoscopic region of interest (ROI) with a 20-GHz sampling rate. We use it to characterize the temporal profile of the photoemitted electron pulses generated in our instrument and find that the electron pulse does not exactly follow the UV pulse profile as commonly believed. Then, we introduce a new two-dimensional (2D) SM-DTEM imaging approach, which applies a tomographic compressed sensing algorithm to recover the spatiotemporal dynamics of an electron pulse passing through a gold cross-grating sample. We demonstrate the recovery of images at 2.7 × 10^9^ fps with a sequence depth (i.e., the number of frames in the movie) of 140 frames in a 50-ns time window of a 3-*μ*m^2^ field of view (FOV) with nanometer-level spatial resolution. In this approach, the reconstruction is achieved by acquiring streak images of different streak speeds and directions, requiring a measurement time of just over 2 min. We conclude with a perspective of further developments necessary to reduce the acquisition time of the 2D SM-DTEM to less than a second and measure a 0D SM-DTEM trace with a single pulse.

## II. RESULTS

### A. Setup and method

**FIG. 1.**
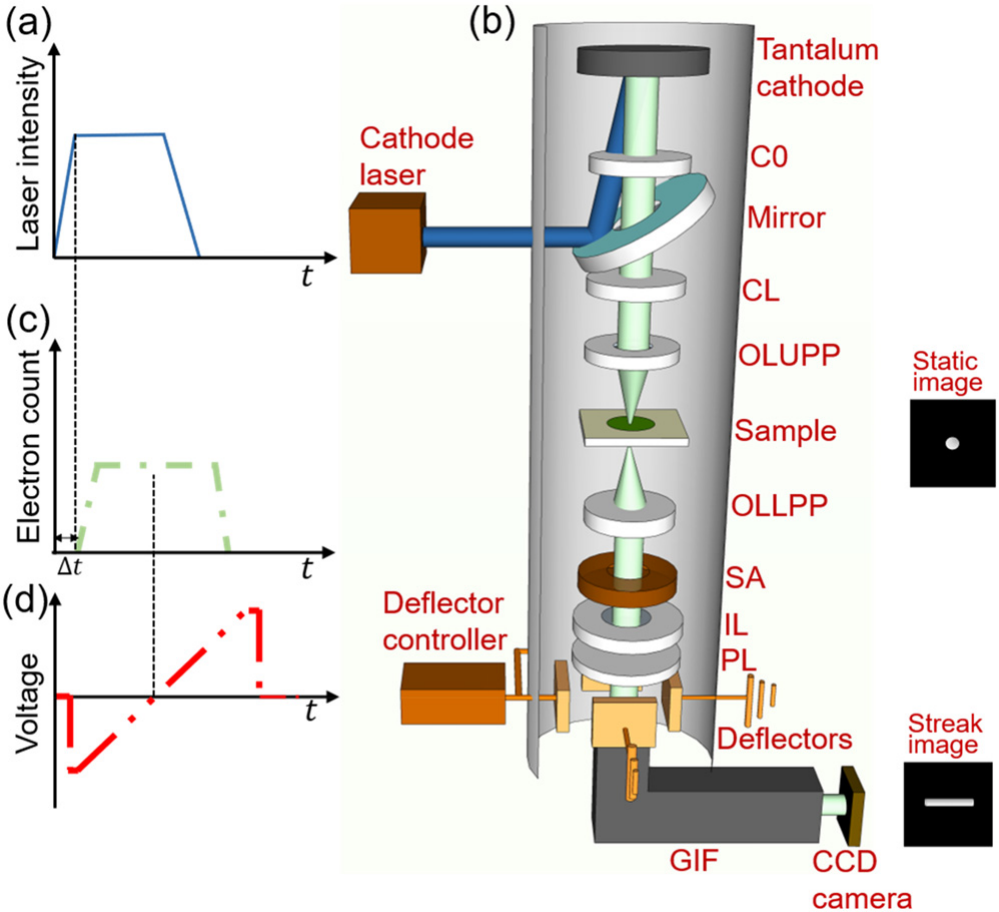
Schematics of the streak-mode dynamic transmission electron microscope (SM-DTEM). (a) Illustration of the cathode laser pulse profile. (b) System configuration of the SM-DTEM. Insets: Illustrations of the static image (top) and the streak image (bottom). C0: Additional condenser lens, CCD: Charge-coupled device; CL: Condenser lens; GIF: Gatan imaging filter; IL: Intermediate lens; OLLPP: Objective lens lower pole piece; OLUPP: Objective lens upper pole piece; PL: Projector lens; SA: Selected-area aperture. (c) Illustration of the photoemitted electron pulse profile. Δt: Time delay. (d) Illustration of the voltage waveform applied to the deflectors.

The SM-DTEM experiments were carried out on the MM-DTEM located at the Institut National de la Recherche Scientifique—Center Énergie Matériaux Télécommunications. This instrument is comprised of an IDES^®^ cathode laser system and a modified Jeol^®^ JEM 2100-Plus TEM.^25^ The laser operates at 10 Hz and allows tuning the pulse duration and profile using an electro-optical modulator to adjust the seed pulse sent into a series of Northrup Grumman^®^ Nd:YAG bulk amplifiers. The output is then sent to a pair of nonlinear crystals to generate fourth harmonic UV light pulses with a wavelength of 266 nm and an energy of up to 1.5 mJ. The cathode laser was configured to produce flat 50-ns UV pulses, as illustrated in Fig. 1(a). The UV light directed into the MM-DTEM column was focused on a Tantalum disk cathode in a Wehnelt with a spacing of 540 *μ*m and a gun accelerating voltage of 200 kV. A schematic diagram of the electron optics of the MM-DTEM is shown in Fig. 1(b). The MM-DTEM has an additional condenser lens (C0) before the standard TEM condenser lens (CL) to compensate for the loss of electrons due to the added laser port.^48^ This design provides the required electron throughput (>10^7^ electrons per pulse) to achieve single-shot imaging. The MM-DTEM also has two pairs of electrostatic deflectors placed after the projector lens, which allows for the collection of multiple frames in an image by deflecting the electron beam using a step voltage waveform from the deflector controller.^25,48^ The beam then passes through a Gatan imaging filter (GIF) and is collected on a charge-coupled device (CCD) camera (2048 × 2048 pixels, each of which is 14 × 14 *μ*m^2^ in size). A flat-top photoemitted electron pulse is best suited for uniform illumination of the sample during an MM-DTEM measurement, as illustrated in Fig. 1(c). The time delay (Δt) shown in the figure is caused by the combination of laser transit to the cathode, photoelectron generation, and photoelectron transit to the deflector plate region. This time delay is compensated in the system control software to synchronize the voltage change of the deflector to the electron pulse and capture the targeted dynamics on the CCD.

The SM-DTEM system has been developed by replacing the step voltage waveform applied to the deflector plates with a voltage ramp [Fig. 1(d)] generated by a customized deflector controller (Axis Photonique Inc.). The voltage ramp is triggered by a 10-Hz TTL signal from the MM-DTEM system controller. When the electron pulse passes through the deflector plates, the trajectories of the electrons are deviated according to the instantaneous voltage, producing a streak image of the FOV across the CCD camera [illustrated in the inset of Fig. 1(b)]. The MM-DTEM contains two pairs of deflector plates so that the photoelectron pulse can be streaked in four directions on the CCD (i.e., leftward, rightward, upward, and downward).

To synchronize the voltage ramp with the photoelectron beam, the time delay was adjusted until the streak image was centered in the CCD for each of the following linear voltage sweep speeds: 4.0, 2.0, 1.3, 1.0, and 0.8 V/ns. Profiles of the electron pulses were then obtained by binning the counts in the image perpendicular to the streak direction. Then, by measuring the change in the position of the streak image on the camera as a function of the deflector plate time delay, a factor was determined to convert the x axis of such streak profiles from units of pixels to nanoseconds. The conversion factors for each voltage sweep speed were found to be 40 pixel/ns, 20 pixel/ns, 13 pixel/ns, 10 pixel/ns, and 8 pixel/ns.

In the following demonstrations of the SM-DTEM imaging capabilities, a Ted Pella^®^ gold cross-grating sample with 2160 line/mm was utilized. The streak images were collected with a 10-s camera exposure, corresponding to the signal accumulation from 100 pulses on the camera. A magnification of 2300× between the sample and the detector was used for all SM-DTEM experiments. The magnification ratio between the object plane and the SA plane was 53.9×, while the magnification ratio from the selected-area aperture (SA) plane to the CCD camera plane was 42.7×.

### B. Demonstration of 0D SM-DTEM

The 0D SM-DTEM measurement strategy involves first placing the SA in the FOV of the static image, collecting a streak image, and then extracting a linear trace along the streak direction of the average transmitted intensity as a function of time. The size of the SA used determines the ROI, as well as the temporal resolution of the measurement. A rough estimate of the temporal resolution of such a measurement is then given by the size of the ROI in pixels multiplied by the streak rate of the image on the camera given in nanoseconds per pixel.

The time to collect a 0D SM-DTEM trace can be as short as a single DTEM pulse, and it directly provides access to dynamics occurring on time scales faster than the pulse duration. As demonstrated by Bostanjoglo *et al.*,^43–46^ a single-shot 0D SM-DTEM measurement is well suited to extract the time scale of laser melting occurring homogeneously in the FOV. Defining a smaller ROI can also allow for its application to measure dynamics happening in more inhomogeneous microstructures, such as composites or polycrystalline materials. Such a measurement may also be used to enhance the time resolution of *in situ* TEM studies of crystal defect creation due to cyclic application of an electric field or pulsed laser heating. In one such experiment, the shear fault density created in a rutile lamella near an electrode tip was found to change with the number of applied voltage cycles.^49^ Therefore, a short measurement time over just a few cycles is critical to uncover the dynamics of such a process.

**FIG. 2.**
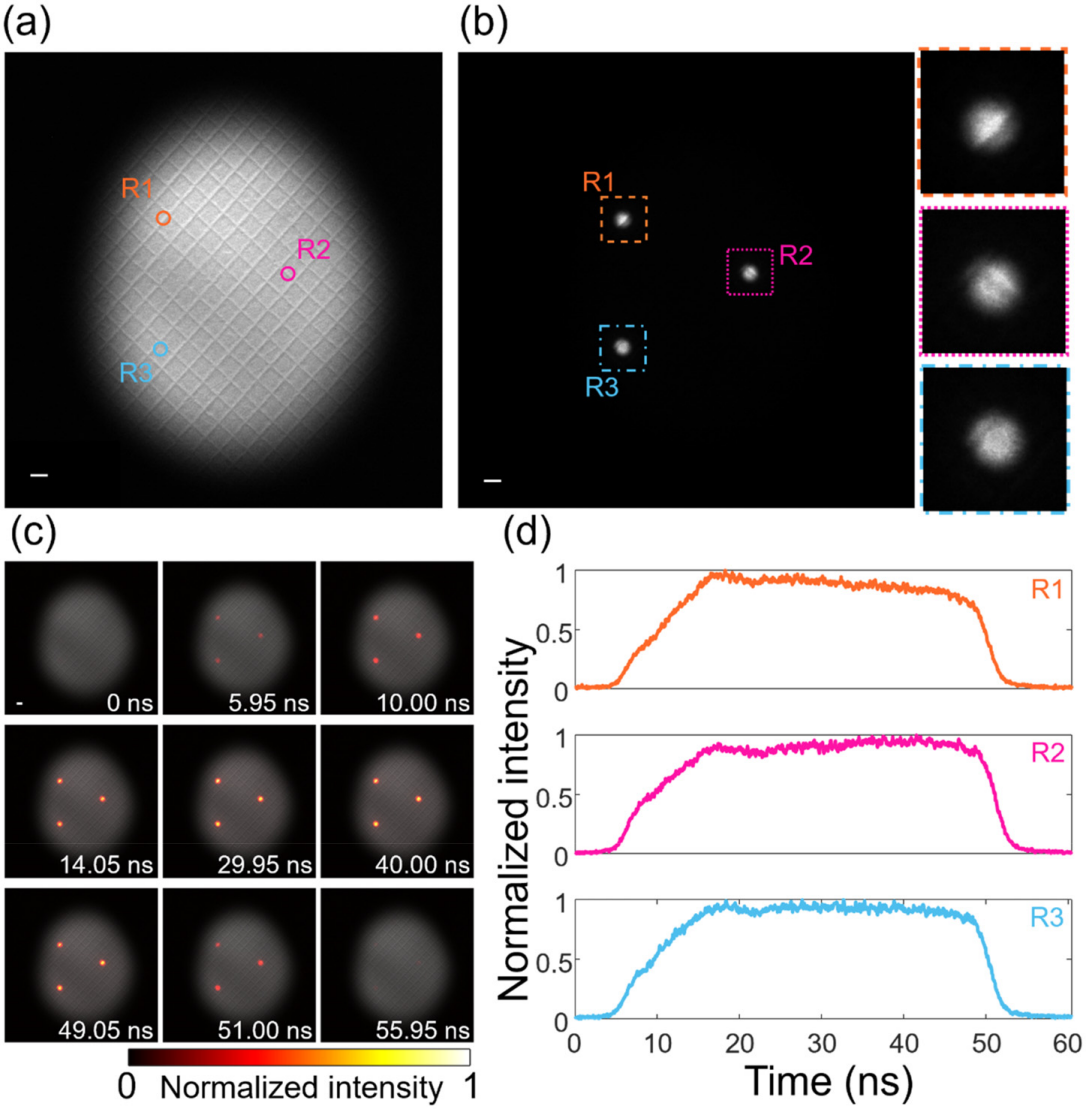
Zero-dimensional (0D) SM-DTEM. (a) Static image of a gold cross-grating sample. (b) Three selected positions constrained by a 20-*μ*m-diameter SA. Right column: Zoomed-in views of the three points. (c) Selected frames of the photoemissions merged to the static image in (a). Scale bar in (a)–(c): 500 nm. (d) Temporal profiles of the three selected regions of interest.

To demonstrate the data quality and capability to selectively define the ROI, we performed a series of measurements on the gold cross-grating sample that was described in Sec. II A. A static image of the full 12.49 × 12.49-*μ*m^2^ FOV is shown in Fig. 2(a). We then employed a 20-*μ*m-diameter SA to define an ROI with a diameter of 370 nm on the sample. A series of streak images were collected by placing the SA at the three different positions indicated in Fig. 2(a) using a 50-ns UV pulse with a 1-mJ pulse energy. The images of the SA were swept from left to right with a speed of 2.0 V/ns, which corresponds to a sampling rate of 20 GHz (i.e., 50 ps per time bin) with 1200 data points in the trace. Using this aperture size and streak rate, we estimated the temporal resolution of the trace to be 3 ns. To better show the capability of the 0D SM-DTEM, a video was generated by merging the data from the three measurements to the static image of the gold cross-grating sample [Fig. 2(b)]. The datacube of the three ROIs was obtained by modulating their static images by the measured temporal profiles and overlaying it on a gray background image of the sample that was later captured with a large FOV. Nine selected frames from the generated video (Visualization 1)^50^ are shown in Fig. 2(c). The measured intensity profiles are also shown in Fig. 2(d), and all profiles show a similar trend in the transmitted intensity. Closer inspection finds a slight peak in the profiles around 18 ns. The profiles slightly deviate at later times, just above the noise of the trace.

### C. Photoemission characterization by 0D SM-DTEM

**FIG. 3.**
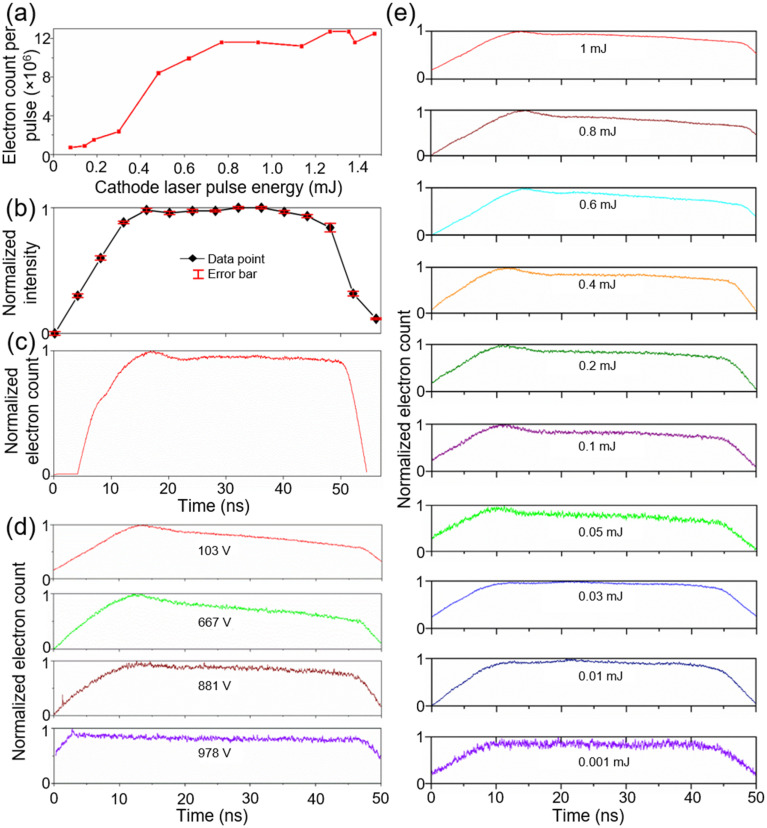
Photoemission characterization using the SM-DTEM. (a) Photoelectron yield vs cathode laser pulse energy. (b) Profile of a 50-ns cathode laser pulse. Error bar: standard deviation. (c) Profile of a 50-ns photoemitted electron pulse. (d) Photoemission profiles with four different Wehnelt biases. (e) Photoemission profiles generated by 10 different cathode laser pulse energies.

We also used 0D SM-DTEM measurements to characterize the temporal profile of the photoemitted electron pulse in more detail. For these measurements, the sample was removed from the column, and streak images were collected with the beam centered in a 100-*μ*m SA at a sweep rate of 4 V/ns, corresponding to an estimated time resolution of 7 ns. These parameters were chosen to maximize the electron counts on the camera as the photoemission pulse profile was found to be rather spatially uniform, as also shown in the measurements presented in Fig. 2. The photoemitted electron yield shown in Fig. 3(a) was determined separately from measurements of the total number of electrons per pulse collected on the camera as a function of UV pulse energy output from the laser. The yield is shown to increase rather linearly until 0.8 mJ when it saturates to a value of around 1.2 × 10^7^ electrons per pulse. This laser output pulse energy corresponds to a UV fluence of 2.3 J/cm^2^ incident on the photocathode based on measurements of a TEM column throughput of 30% and UV spot size of 115 *μ*m.

The normalized temporal pulse profile of the 50-ns cathode UV laser pulse used for the measurements is shown in Fig. 3(b). This profile was measured with a silicon-amplified photodiode with a 2-ns time resolution at a point along the beam path before entering the microscope. A representative temporal profile of the current in a laser-generated electron pulse is shown in Fig. 3(c). This trace was taken from a streak image accumulated over 100 pulses using a laser pulse energy of 1 mJ and a Wehnelt bias of 103 V. Interestingly, a peak in the pulse profile is seen at around 18 ns, after which the electron count decreases by 6% and then remains flat for the remainder of the pulse. It is also interesting to note that the simulation of photoemitted electron trajectories of similar gun configurations has found that the cathode-to-anode transit time is also in the range of a few nanoseconds, which is the same time scale as the observed peak in the pulse profile.^24^

A series of further measurements were conducted to investigate the cause of the unexpected peak in the photoelectron profile. Keeping the UV pulse energy at 1 mJ, streak images were collected with increasing Wehnelt bias voltages. The Wehnelt bias setting was converted into an approximate voltage using a lookup table provided by the TEM manufacturer. The Wehnelt filters photoemitted electrons by focusing them into a virtual source within the electron gun.^23^ Electrons with dramatically different energy or those that do not follow a straight trajectory originating from the center of the cathode do not fall into this focus. They are later screened by the electron gun anode aperture. Figure 3(d) shows that the measured electron pulse profiles are susceptible to the applied Wehnelt voltages and that increasing it leads to a flatter pulse. The peak is still clearly visible when applying a voltage of 667 V to the Wehnelt but disappears for 881 V and higher. This measurement suggests that the observed peak in the pulse profile comes from electron interactions in the electron gun, not elsewhere in the microscope column. The electrons contributing to the peak have an energy or trajectory that deviates from the ideal values.

Then, the electron pulse profile was measured as a function of the UV laser pulse energy, as shown in Fig. 3(e). It is seen that with decreasing pulse energy, the peak in the electron pulse gradually shifts from 18 to 10 ns; however, it is found to persist until the UV pulse energy is reduced to 0.03 mJ. While this pulse profile is desirable for more uniform illumination of the sample, the electron yield at this pulse energy is less than 10^6^ electrons per pulse, which makes it too weak for single-shot imaging. Cross-referencing this information with the trend in Fig. 3(a), it is seen that the peak in the photoemission persists for electron charge densities that are an order of magnitude below the photoemission saturation value. This finding suggests that electron screening inside the electron gun (Child–Langmuir effect) is not responsible for this feature in the electron pulse profile. Furthermore, investigations are ongoing to test other explanations for the observed peak in the photoemission trace, including the generation of an opposing inductive voltage caused by the fast current rise time of the electron pulse^51,52^ or laser ablation of the Ta cathode.^53^

### D. Demonstration of 2D SM-DTEM imaging

Despite allowing straightforward measurements of contrast changes within a pulse duration, the 0D SM-DTEM has the major limitation that achieving a faster temporal resolution requires reducing the size of the SA. In turn, this shortcoming limits the spatial content and electron throughput of the measurement. As a consequence, this approach requires a large number of measurements via point scanning to obtain a full 2D image of the sample.

In the following, we will show that 2D SM-DTEM imaging can overcome these challenges and enable high-throughput spatiotemporal imaging based on compressed ultrafast tomographic imaging (CUTI).^54^ Integrating the concept of sparse-view computed tomography to the spatiotemporal domain, CUTI employs temporal shearing and spatiotemporal integration to achieve passive projections of the transient phenomena. Particularly, a CUTI system includes three essential units: an imaging unit, a temporal shearing unit, and a detector. After being imaged by the imaging unit, an ( x, y, t) datacube is spatiotemporally tilted to a specific angle. The detector passively integrates the tilted datacube to a 2D image, which is equivalent to a passive spatiotemporal projection of the event from a particular angle. Utilizing the multiple sweeping speeds and ranges of typical temporal shearing units (e.g., streak cameras), passive projections at various spatiotemporal angles can be acquired. Eventually, through the implementation of a compressed sensing-based reconstruction algorithm, the ( x, y, t) datacube of the transient event can be effectively reconstructed from a few streak images.

**FIG. 4.**
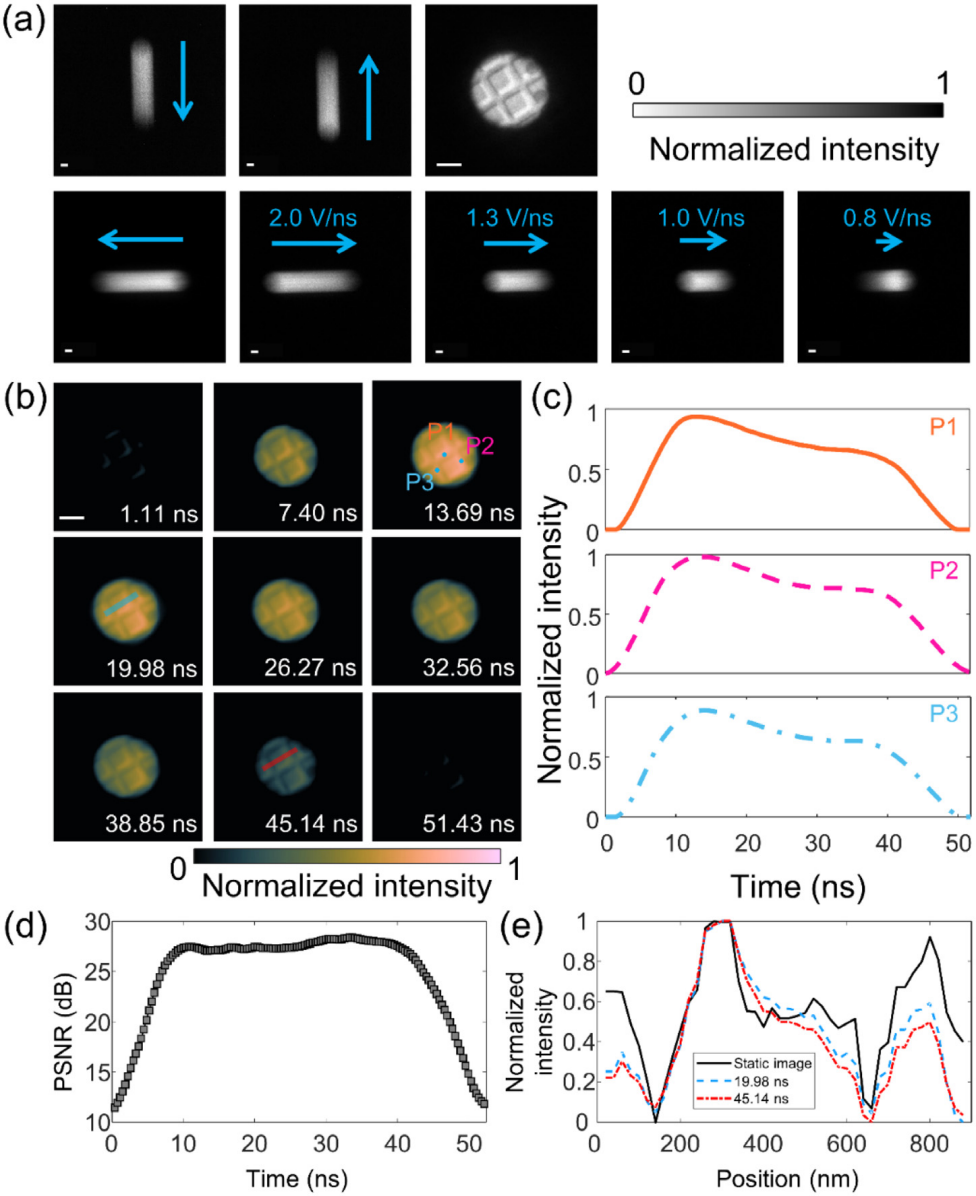
Two-dimensional (2D) SM-DTEM enabled by compressed ultrafast tomographic imaging. (a) Static image of a gold cross-grating constrained by a 200-*μ*m-diameter SA and seven representative streak images under different shearing conditions and directions. Arrow orientation: shearing direction; Arrow length: shearing speeds. (b) Selected frames of the reconstruction. Scale bars in (a) and (b): 500 nm. (c) Temporal profiles of three selected points marked in (b). (d) Peak signal-to-noise ratio (PSNR) of the reconstructed images as a function of time after the pulse onset. (e) Selected intensity cross sections of the static image and reconstructed grating images at 19.98 and 45.14 ns. The locations used to generate these cross sections are, respectively, indicated in (b) as blue and red lines.

To demonstrate the 2D SM-DTEM, we studied the transmission of a 50-ns electron pulse through the gold cross-grating sample used in the experiments described in Sec. II B. A 100-*μ*m-diameter SA was used, which defined a 1.85-*μ*m-diameter 2D FOV on the sample plane. Based on the multi-directional and multi-scale capabilities of the deflectors in the SM-DTEM system, 17 projections were acquired, including one static image and 16 streak images of four sweeping speeds (2.0, 1.3, 1.0, and 0.8 V/ns) in four directions [Fig. 4(a)]. The acquired measurements were input into a two-step iterative shrinkage/thresholding (TwIST) algorithm-based tomographic reconstruction (TTR) algorithm^55,56^ to recover the transient event. Details of the raw data treatment, as well as a review of the TwIST algorithm and its implementation, are provided in supplementary material Sec. S1.^50^ The TTR algorithm recovered the datacube of the dynamic scene with an imaging speed of ∼2.7 × 10^9^ fps (i.e., a 370-ps inter-frame interval) and a sequence depth of 140 frames (Visualization 2).^50^ It should be noted that this inter-frame interval provides more than an order of magnitude improvement over the 7-ns time resolution 0D SM-DTEM measurements made with the same size SA in Sec. II C. Figure 4(b) presents nine selected frames of the CUTI-reconstructed results, which highlight the clarity of the spatial features in the reconstructed images. Notably, the square grid of the grating is resolved, as is the nonuniform contrast gradient observable within a square. Closer inspection finds two dark circles near the right edge of FOV, which are latex spheres decorating the surface of the gold grating. These results demonstrate that the 2D SM-DTEM can recover subtle changes in contrast.

The intensity profiles of three selected positions [marked in Fig. 4(b)] are presented in Fig. 4(c). A peak in the profiles is found around 15 ns, again in agreement with the previously presented measurements in Secs. II B and II C. The similarity of each profile shows the homogeneity of the sample region. Also, it shows the capability of 2D mode to extract the intensity profile over the entire FOV, providing information about the nature of the sample. To evaluate the TTR algorithm performance, we calculated the peak signal-to-noise ratio (PSNR) of the recovered images. As shown in Fig. 4(d), the reconstructed images recovered within the intense region of the pulse (between 7 and 43 ns) exhibited good quality with a PSNR > 25. At the beginning and end of the pulse, the signal was reduced by an order of magnitude or more, corresponding to a significantly lower PSNR. Moreover, to investigate the recovered contrast, we extracted intensity cross sections from images corresponding to 19.98 and 45.14 ns along a line that traversed the central grating square [Fig. 4(e)]. Compared with the static image, the average errors of these cross sections were calculated to be 14.9% and 17.3%, respectively, suggesting that the recovered contrast was well conserved allowing for easy identification of the features in the images.

We also carried out a simulation of a more complex dynamic scene to test the performance of the algorithm to image a sample that is rapidly changing under the electron beam. The scene consists of three balls that change position and size during a datacube of 20 frames of a 128 × 128 pixel FOV. Streak images were then simulated and reconstructed using the TTR algorithm. The simulation and reconstruction code are described in supplementary material Sec. S2.^50^ A movie comparing the ground truth and reconstruction is available in Visualization 3.^50^ Five frames of the ground truth and recovered scene are shown in Fig. S2,^50^ depicting good agreement in the size and position of all features in the scene. Furthermore, the centroid and size of each object in the recovered scene are also shown to follow the known values of the simulation. Therefore, we believe that this method is well suited to image scenes where the sample morphology is also evolving, and we are in the process of carrying out an experimental demonstration. We have deposited the simulation and TTR algorithm source code in an open repository (supplementary material Ref. S33)^50^ to allow anyone to test it on other dynamic scenes of interest and contribute to its development.

## III. DISCUSSION

A number of improvements are envisioned to optimize the time necessary to collect a set of streak images used in the presented 2D SM-DTEM method. Each of the 16 streak images in the dataset shown in Sec. II D was acquired by manually changing streak speeds and streak directions before collecting an image. Software and slight hardware modifications to automate this process are expected to reduce the measurement time of a full dataset to under 3 min, and this process can be further improved by increasing the repetition rate of the electron pulse generation. Furthermore, as in the MM-DTEM, multiple streak images can be collected at different positions on the camera before they are read out. Therefore, a system producing the same electron counts per pulse operating at 6 kHz, collecting four streak images on a camera that are readout at 15 fps, can collect the same dataset in just 0.24 s. Increasing the electron counts per pulse reduces the necessary repetition rate of the laser but will not significantly improve the dataset measurement time, which is mainly limited by the number of streak images that can be placed on the camera and its readout time. However, developing algorithms or data collection strategies that require less than 16 streak images in a dataset without sacrificing the reconstructed time series data quality will further proportionately reduce the data collection time.

Reducing the number of shots averaged to form a streak image is important for studying dynamics sensitive to the number of excitation cycles. Acquiring a high-quality streak image with a single shot also allows for the collection of 0D SM-DTEM traces of irreversible dynamics occurring within the electron pulse duration. From the measurements presented here, it is seen that achieving this goal requires increasing the photoelectron yield of our instrument by a factor of 10–100 to more than 10^8^ electrons per pulse. A number of investigations are planned toward this goal, including studies of the tradeoffs with laser pulse duration, spot size, and photocathode material while characterizing the resulting spatiotemporal pulse profile with the developed SM-DTEM modalities.

## IV. CONCLUSION

In summary, we have presented two SM-DTEM operational modes and used them to study the spatiotemporal profile of photoemitted electron pulses. First, the 0D SM-DTEM was shown to provide a direct measurement of the average contrast evolution in a desired ROI with a time resolution faster than the pulse duration. Collected traces with time resolutions of 3–7 ns were used to demonstrate that the DTEM photoemitted electron pulse did not strictly follow the input UV laser pulse. Specifically, a peak was found between 10 and 18 ns that unexpectedly persisted for pulse energies well below the photoemission saturation limit. Second, 2D SM-DTEM imaging was demonstrated, incorporating the CUTI paradigm for efficient ultrafast imaging using existing DTEM hardware. Notably, we were able to reconstruct detailed images with a 370-ps inter-frame interval and a 140-frame depth in a 50-ns time window. The reconstructed images in this proof of principle demonstration were shown to have good quality and allow for easy identification of the features in the scene. Further experimental work is necessary to determine the performance of this method with respect to signal level, contrast mechanisms, and increased complexity of sample dynamics. Finally, the time to measure a 2D SM-DTEM dataset can be decreased significantly with continued instrumentation development, alleviating the sample stability constraint and enabling the study of picosecond dynamics of materials as they evolve.

## Data Availability

The data that support the findings of this study are available from the corresponding author upon reasonable request.

## References

[1] W. E. King , M. R. Armstrong , O. Bostanjoglo , and B. W. Reed , in *Science of Microscopy*, edited by P. W. Hawkes and J. C. H. Spence ( Springer, New York, 2007), pp. 406–444.

[2] P. Moradifar , Y. Liu , J. Shi , M. L. S. Thurston , H. Utzat , T. B. van Driel , A. M. Lindenberg , and J. A. Dionne , arXiv:2212.10099 (2022). 10.1021/acs.chemrev.2c00917 37979189

[3] F. M. Alcorn , P. K. Jain , and R. M. van der Veen , Nat. Rev. Chem. 7(4), 256–272 (2023). 10.1038/s41570-023-00469-y 37117417

[4] M. S. Grinolds , V. A. Lobastov , J. Weissenrieder , and A. H. Zewail , Proc. Natl. Acad. Sci. 103(49), 18427–18431 (2006). 10.1073/pnas.0609233103 17130445 PMC1693681

[5] S. T. Park , D. J. Flannigan , and A. H. Zewail , J. Am. Chem. Soc. 134(22), 9146–9149 (2012). 10.1021/ja304042r 22591381

[6] R. M. Van Der Veen , O.-H. Kwon , A. Tissot , A. Hauser , and A. H. Zewail , Nat. Chem. 5(5), 395–402 (2013). 10.1038/nchem.1622 23609090

[7] J. Chapman , P. Batson , E. Waddell , and R. Ferrier , Ultramicroscopy 3, 203–214 (1978). 10.1016/S0304-3991(78)80027-8 358526

[8] U. J. Lorenz and A. H. Zewail , Proc. Natl. Acad. Sci. 110(8), 2822–2827 (2013). 10.1073/pnas.1300630110 23382239 PMC3581949

[9] V. A. Lobastov , R. Srinivasan , and A. H. Zewail , Proc. Natl. Acad. Sci. 102(20), 7069–7073 (2005). 10.1073/pnas.0502607102 15883380 PMC1129142

[10] O. Bostanjoglo and D. Otte , Mater. Sci. Eng.: A 173(1–2), 407–411 (1993). 10.1016/0921-5093(93)90254-C

[11] E. Ma , C. Thompson , L. Clevenger , and K.-N. Tu , Appl. Phys. Lett. 57(12), 1262–1264 (1990). 10.1063/1.103504

[12] O. Bostanjoglo and D. Otte , Phys. Status Solidi A 150(1), 163–169 (1995). 10.1002/pssa.2211500114

[13] O. Bostanjoglo and F. Heinricht , Rev. Sci. Instrum. 61(4), 1223–1229 (1990). 10.1063/1.1141952

[14] T. Nink , Z. Mao , and O. Bostanjoglo , Appl. Surf. Sci. 154, 140–145 (2000). 10.1016/S0169-4332(99)00438-9

[15] H. Kleinschmidt , A. Ziegler , G. Campbell , J. Colvin , and O. Bostanjoglo , J. Appl. Phys. 98(5), 054313 (2005). 10.1063/1.2035899

[16] T. Nink , F. Galbert , Z. Mao , and O. Bostanjoglo , Appl. Surf. Sci. 138, 439–443 (1999). 10.1016/S0169-4332(98)00438-3

[17] G. H. Campbell , T. B. LaGrange , W. E. King , J. D. Colvin , A. Ziegler , N. D. Browning , H. Kleinschmidt , and O. Bostanjoglo , “The Hcp to Bcc phase transformation in Ti characterized by nanosecond electron microscopy,” in *Solid-Solid Phase Transformations in Inorganic Materials*, edited by J. M. Howe, J. K. Lee, D. E. Laughlin, D. J. Srolovitz, and U. Dahmen (TMS, Warrendale, PA, 2005).

[18] M. R. Armstrong , K. Boyden , N. D. Browning , G. H. Campbell , J. D. Colvin , W. J. DeHope , A. M. Frank , D. J. Gibson , F. Hartemann , and J. S. Kim , Ultramicroscopy 107(4–5), 356–367 (2007). 10.1016/j.ultramic.2006.09.005 17169490

[19] T. LaGrange , M. Armstrong , K. Boyden , C. Brown , G. Campbell , J. Colvin , W. DeHope , A. Frank , D. Gibson , and F. Hartemann , Appl. Phys. Lett. 89(4), 044105 (2006). 10.1063/1.2236263

[20] I. Langmuir , Phys. Rev. 2(6), 450 (1913). 10.1103/PhysRev.2.450

[21] Y. Kawamura , Y. U. Jeong , Y. Akiyama , S. Kubodera , K. M. K. Midorikawa , and K. T. K. Toyoda , Jpn. J. Appl. Phys., Part 2 32(2B), L297 (1993). 10.1143/JJAP.32.L297

[22] E. Kieft , K. B. Schliep , P. K. Suri , and D. J. Flannigan , Struct. Dyn. 2(5), 051101 (2015). 10.1063/1.4930174 26798820 PMC4711641

[23] K. Bücker , M. Picher , O. Crégut , T. LaGrange , B. Reed , S. Park , D. Masiel , and F. Banhart , Ultramicroscopy 171, 8–18 (2016). 10.1016/j.ultramic.2016.08.014 27584052

[24] S. Ji , L. Piazza , G. Cao , S. T. Park , B. W. Reed , D. J. Masiel , and J. Weissenrieder , Struct. Dyn. 4(5), 054303 (2017). 10.1063/1.4994004 28781982 PMC5515673

[25] T. LaGrange , B. W. Reed , and D. J. Masiel , MRS Bull. 40(1), 22–28 (2015). 10.1557/mrs.2014.282

[26] T. LaGrange , B. Reed , W. DeHope , R. Shuttlesworth , and G. Huete , Microsc. Microanal. 17(S2), 458–459 (2011). 10.1017/S1431927611003163

[27] K. Song , L. Liu , D. Zhang , M. P. Hautzinger , S. Jin , and Y. Han , Adv. Energy Mater. 10(26), 1904006 (2020). 10.1002/aenm.201904006

[28] D. Zhang , Y. Zhu , L. Liu , X. Ying , C.-E. Hsiung , R. Sougrat , K. Li , and Y. Han , Science 359(6376), 675–679 (2018). 10.1126/science.aao0865 29348363

[29] E. J. VandenBussche , C. P. Clark , R. J. Holmes , and D. J. Flannigan , ACS Omega 5(49), 31867–31871 (2020). 10.1021/acsomega.0c04711 33344840 PMC7745440

[30] T. Mashimo , A. Nakamura , and Y. Hamada , SPIE Proc. 1801, 170–175 (1993). 10.1117/12.145764

[31] R. Krishnan , H. Saitoh , H. Terada , V. Centonze , and B. Herman , Rev. Sci. Instrum. 74(5), 2714–2721 (2003). 10.1063/1.1569410

[32] A. Velten , A. Schmitt-Sody , J.-C. Diels , S. Rostami , A. Rasoulof , C. Feng , and L. Arissian , J. Phys. B 48(9), 094020 (2015). 10.1088/0953-4075/48/9/094020

[33] D. Qi , S. Zhang , C. Yang , Y. He , F. Cao , J. Yao , P. Ding , L. Gao , T. Jia , J. Liang , Z. Sun , and L. V. Wang , Adv. Photonics 2(1), 014003 (2020). 10.1117/1.AP.2.1.014003

[34] See https://www.hamamatsu.com/eu/en/product/photometry-systems/streak-camera/universal-streak-camera.html for information about commercial streak cameras with a temporal resolution at hundreds of femtoseconds.

[35] See https://www.axis-photon.com/ultrafast-streak-cameras for information about commercial streak cameras with a temporal resolution at hundreds of femtoseconds.

[36] M. Marquez , Y. Lai , X. Liu , C. Jiang , S. Zhang , H. Arguello , and J. Liang , IEEE J. Sel. Top. Signal Process. 16(4), 688–699 (2022). 10.1109/JSTSP.2022.3172592

[37] Y. Lai , Y. Xue , C. Y. Côté , X. Liu , A. Laramée , N. Jaouen , F. Légaré , L. Tian , and J. Liang , Laser Photonics Rev. 14(10), 2000122 (2020). 10.1002/lpor.202000122

[38] I. Yagi , S. Okada , T. Matsumoto , D. Wang , T. Namihira , and K. Takaki , IEEE Trans. Plasma Sci. 39(11), 2232–2233 (2011). 10.1109/TPS.2011.2154386

[39] J. Liang , P. Wang , L. Zhu , and L. V. Wang , Nat. Commun. 11(1), 5252 (2020). 10.1038/s41467-020-19065-5 33067438 PMC7567836

[40] P. Ding , Y. Yao , D. Qi , C. Yang , F. Cao , Y. He , J. Yao , C. Jin , Z. Huang , and L. Deng , Adv. Photonics 3(4), 045001 (2021). 10.1117/1.AP.3.4.045001

[41] X. Liu , J. Liu , C. Jiang , F. Vetrone , and J. Liang , Opt. Lett. 44(6), 1387–1390 (2019). 10.1364/OL.44.001387 30874657

[42] D. Faccio and A. Velten , Rep. Prog. Phys. 81(10), 105901 (2018). 10.1088/1361-6633/aacca1 29900876

[43] O. Bostanjoglo and T. Nink , J. Appl. Phys. 79(11), 8725–8729 (1996). 10.1063/1.362499

[44] O. Bostanjoglo and J. Kornitzky , in *Proceedings of 12th International Congress on Electron Microscopy* (San Francisco Press, San Francisco, 1990), Vol. 1, p. 180.

[45] O. Bostanjoglo , J. Kornitzky , and R. Tornow , J. Appl. Phys. 69(4), 2581–2583 (1991). 10.1063/1.348647

[46] O. Bostanjoglo and T. Nink , Appl. Surf. Sci. 109, 101–105 (1997). 10.1016/S0169-4332(96)00912-9

[47] X. Liu , S. Zhang , A. Yurtsever , and J. Liang , Micron 117, 47–54 (2019). 10.1016/j.micron.2018.11.003 30472498

[48] T. LaGrange , G. H. Campbell , B. Reed , M. Taheri , J. B. Pesavento , J. S. Kim , and N. D. Browning , Ultramicroscopy 108(11), 1441–1449 (2008). 10.1016/j.ultramic.2008.03.013 18783886

[49] R. J. Kamaladasa , A. A. Sharma , Y.-T. Lai , W. Chen , P. A. Salvador , J. A. Bain , M. Skowronski , and Y. N. Picard , Microsc. Microanal. 21(1), 140–153 (2015). 10.1017/S1431927614013555 25529361

[50] See the supplementary material online for supplementary material Sec. S1: 2D SM-DTEM TTR implementation and data analysis. Supplementary material Sec. S2: Simulation of complex dynamic scene and TTR algorithm. Visualization 1: 0D SM-DTEM measurements of a gold cross-grating sample. Three ROIs were overlayed on a gray background image of the sample. Visualization 2: Reconstructed scene of a gold cross-grating sample measured by 2D SM-DTEM. The video was reconstructed using the TTR algorithm. Visualization 3: Simulated dynamic scene of three balls with varying positions and sizes. The ground truth and reconstruction were compared.

[51] P. Zhang , Á. Valfells , L. Ang , J. Luginsland , and Y. Lau , Appl. Phys. Rev. 4(1), 011304 (2017). 10.1063/1.4978231

[52] J. W. Luginsland , S. McGee , and Y. Lau , IEEE Trans. Plasma Sci. 26(3), 901–904 (1998). 10.1109/27.700866

[53] S. Mittelmann , J. Oelmann , S. Brezinsek , D. Wu , H. Ding , and G. Pretzler , Appl. Phys. A 126, 672 (2020). 10.1007/s00339-020-03838-2

[54] Y. Lai , R. Shang , C.-Y. Côté , X. Liu , A. Laramée , F. Légaré , G. P. Luke , and J. Liang , Opt. Lett. 46(7), 1788–1791 (2021). 10.1364/OL.420737 33793544 PMC8050836

[55] J. M. Bioucas-Dias and M. A. Figueiredo , IEEE Trans. Image Process. 16(12), 2992–3004 (2007). 10.1109/TIP.2007.909319 18092598

[56] Y. Lai , R. Shang , C.-Y. Côté , X. Liu , A. Laramée , in F. Légaré , G. P. Luke , and J. Liang , Proc. SPIE 12008, 120080S (2022). 10.1117/12.2608938

